# Rise of multidrug-resistant non-vaccine serotype 15A *Streptococcus pneumoniae* in the United Kingdom, 2001 to 2014

**DOI:** 10.2807/1560-7917.ES.2016.21.50.30423

**Published:** 2016-12-15

**Authors:** Carmen Sheppard, Norman K. Fry, Shazad Mushtaq, Neil Woodford, Rosy Reynolds, Regina Janes, Rachel Pike, Robert Hill, Maimuna Kimuli, Peter Staves, Michel Doumith, Timothy Harrison, David M Livermore

**Affiliations:** 1 Respiratory and Vaccine Preventable Bacteria Reference Unit, Public Health England, London, United Kingdom; 2 Antimicrobial Resistance and Healthcare Associated Infections Reference Unit, Public Health England, London, United Kingdom; 3 Norwich Medical School, University of East Anglia, Norwich, United Kingdom; 4 Southmead Hospital, Bristol, United Kingdom; 5 British Society for Antimicrobial Chemotherapy, Birmingham, United Kingdom; 6 LGC, Fordham, United Kingdom

**Keywords:** Pneumococcal conjugate vaccine, *Streptococcus pneumoniae*, Capsular serotypes

## Abstract

Conjugate vaccines have reduced pneumococcal disease in vaccinated children and unvaccinated adults, but non-vaccine serotypes are of concern, particularly if antibiotic resistant. We reviewed *Streptococcus pneumoniae* collected via: (i) the British Society for Antimicrobial Chemotherapy (BSAC) surveillances from 2001–2014; (ii) Public Health England’s (PHE) invasive isolate surveillance from 2005–2014 and (iii) referral to PHE for resistance investigation from 2005–2014. Serotype 15A increased in all series, with many representatives showing triple resistance to macrolides, tetracyclines and penicillin. 15A was consistently among the 10 most prevalent serotypes from 2011 in PHE and BSAC invasive isolate/bacteraemia surveillance but never previously; 26–33% of these invasive 15A isolates had triple resistance. BSAC respiratory isolates were only serotyped in 2013/14 and 2014/15 (October to September); 15A was most prevalent serotype in both periods, comprising 9–11% of isolates, 38–48% of them with triple resistance. Serotype 15A represented 0–4% of *S. pneumoniae* referred to PHE for reference investigation annually until 2008 but rose to 29% (2013) and 32% (2014). Almost all multidrug-resistant 15A isolates were sequence type (ST) 63 variants, whereas susceptible 15A isolates were clonally diverse. The rise of serotype 15A suggests that pneumococcal conjugate vaccines will need ongoing adaptation.

## Introduction

Seven-valent pneumococcal conjugate vaccine (Prevenar 7, PCV7) first became available internationally in 2000, and protects against invasive *Streptococcus pneumoniae* infection by serotypes 4, 6B, 9V, 14, 18C, 19F and 23F. Numerous countries have reported that deployment reduced the incidence of invasive (i.e. blood and cerebrospinal fluid (CSF)) *S. pneumoniae* disease both in children, who are vaccinated, and in elderly adults, who benefit through reduced carriage and transmission of virulent serotypes by children [1-4]. Antibiotic resistance was historically concentrated in five PCV7-targeted serotypes (6B, 9V, 14, 19F and 23F) [5] and several countries have reported reductions in the prevalence of resistance as these were displaced [6]. United Kingdom (UK) experience conforms to these general patterns [7], with the caveat that penicillin-non-susceptible *S. pneumoniae* were uncommon before the vaccine’s introduction to the childhood schedule in 2006/07, meaning that little further fall occurred; macrolide resistance was reduced, reflecting displacement of a resistant serotype 14 lineage [8,9].

The success of PCV7 was partly offset by rises in other serotypes; notably 19A, where multidrug resistance to antibiotics became frequent [10,11]. This was countered by replacing PCV7 with a 13-valent conjugate vaccine (PCV13), additionally covering serotypes 1, 3, 5, 6A, 7F and 19A. PCV13 replaced PCV7 in the UK in April 2010 and this switch was followed by (i) reduced infant carriage of these additional serotypes [12], and (ii) a further 56% reduction in invasive disease incidence from a post-PCV7 baseline [13]. Again, however, rises are being seen in other, non-vaccine, serotypes, principally 8, 10A, 12F, 15A and 24F [13]. Serotype 15A is of particular interest since multidrug-resistant isolates belonging to this serotype have been reported as far apart as east Asia [14-16], North America [17,18], Norway [19], Italy [20] and Australia [21]. Here, we explore the rise of serotype 15A *S. pneumoniae* in the UK and Ireland at epidemiological and molecular levels, using data from both the British Society for Antimicrobial Chemotherapy (BSAC) and Public Health England (PHE) surveillances.

## Methods

### British Society for Antimicrobial Chemotherapy surveillance

The BSAC Bacteraemia and Respiratory Surveillance Programmes have been described previously [22-24]. Both collect isolates from across the UK and Republic of Ireland. The Bacteraemia programme runs on the calendar year. Until 2009 we asked participating laboratories to send up to 10 consecutive bloodstream *S. pneumoniae* isolates per annum from each of 25 hospital laboratories; from 2010 we have similarly sought seven consecutive bloodstream isolates per annum from each of 40 hospital laboratories. Isolates have been serotyped throughout, and results were reviewed across the years 2001 to 2014, inclusive. The Respiratory Programme runs on an October–September year, designated e.g. 2013/14, so that isolates from each winter peak of respiratory disease are not split between calendar years. It examines consecutive isolates from lower respiratory tract infections (LRTIs) in non-hospitalised patients or those hospitalised for less than 48 hours. Until 2013/14 the BSAC Respiratory Surveillance Programme did not routinely serotype isolates, therefore only 2013/14 and 2014/15 data were reviewed. In both these years the surveillance sought 14 consecutive LRTI *S. pneumoniae* isolates from each of the same 40 laboratories contributing to the Bacteraemia surveillance. Actual numbers of isolates collected in both surveillances were somewhat below these targets (see Results) and, in most years, one or two recruited laboratories failed to collect, and were subsequently dropped and replaced by alternative sites. Hospital laboratory mergers, mostly in the past 5 years, have also meant that participating microbiology laboratories increasingly source isolates from multiple hospitals, augmenting representativeness.

### Public Health England invasive isolate surveillance

PHE Colindale routinely seeks submission of all invasive (i.e. blood and CSF) *S. pneumoniae* from hospital laboratories in England, Wales and Northern Ireland, receiving ca 4,000–5,000 isolates each year), over 95% of them from blood. Results of this surveillance were reviewed from 2005 (i.e. 1 year before PCV7 was introduced) to 2014. Susceptibility testing is performed on a subset of these isolates, comprising all those from laboratories contributing to European Antimicrobial Resistance Surveillance (EARS)-net [25]; this total fluctuated between 1,159 and 2,066 organisms annually over the study period.

### Public Health England reference laboratory submissions

Besides surveillance isolates from invasive infections, PHE receives variable numbers of *S. pneumoniae* as reference submissions from respiratory and other non-sterile sites, principally eye and ear infections. Most are sent for investigation because the sender perceives them to have unusual resistance patterns, although senders’ definitions of unusual vary and may be contingent on the site of the infection. Over 95% of isolates are from laboratories in England, Wales and Northern Ireland, with the remaining ca 5% largely from Scotland and the Republic of Ireland. Data were reviewed across the period 2005 to 2014.

### Identification, serotyping and susceptibility testing

All surveillance and referred isolates were confirmed as forming alpha-haemolytic colonies on horse blood agar and being inhibited by a 5 µg optochin (ethylhydrocupreine hydrochloride) disc (Oxoid-Thermofisher, Basingstoke, UK). Isolates with atypical colonial morphology, or which could not be serotyped (below), were confirmed as being lysed within 30 min by 2% sodium deoxycholate, and being catalase-negative when tested with 3% hydrogen peroxide. For serotyping, isolates were grown overnight in Todd Hewitt broth at 35 °C with 5% CO_2_, harvested by centrifugation at 453 *g* for 30 min, then re-suspended in a small residual volume of broth and subjected to slide agglutination tests with standard antisera (Statens Serum Institut, Copenhagen, Denmark) [26]. Agar dilution susceptibility tests were performed in accordance with BSAC guidelines [27], using IsoSensitest agar (Oxoid-Thermofisher) supplemented with 5% defibrinated horse blood and incubated at 35–37 °C in a 5% CO_2_ atmosphere. ‘Triple resistance’ was defined as resistant to erythromycin (minimum inhibitory concentration (MIC) > 0.5 mg/L) and tetracycline (MIC > 2 mg/L), and non-susceptible to penicillin (MIC > 0.06 mg/L), based on EUCAST breakpoints [28])

### DNA extraction, sequencing and bioinformatic analysis

Isolates were grown on horse blood agar (PHE Media Services) and treated by the Qiagen-recommended method for lysis of Gram-negative bacteria (Qiagen, Manchester, UK), which is effective for *S. pneumoniae* and simpler than the Gram-positive protocol. DNA was extracted from the lysates using a QIAsymphony SP automated instrument (Qiagen) and a QIAsymphony DSP DNA Mini Kit, using a tissue extraction protocol. DNA concentrations were measured using the Quant-IT Broad Range DNA Kit (Life Technologies, Paisley, UK) and GloMax 96 Microplate Luminometer (Promega, Southampton, UK). After adjusting to a concentration of 10–30 ng/μl, DNA was sent for whole genome sequencing (WGS) by Illumina methodology. The resulting data were automatically analysed using a bespoke bioinformatic pipeline for *S. pneumoniae*, developed by PHE. Among other things, this (i) checks species identification by a kmer method and (ii) automatically assigns MLST sequence types (STs), identified by mapping the reads against all *S. pneumoniae* allele variants held in the MLST database [29], using a modification of the short-read sequence typing (SRST) software [30]. Resistance genes affecting susceptibility for macrolides and tetracyclines were identified, and their sequences reviewed.

## Results

### Serotype trends, British Society for Antimicrobial Chemotherapy bacteraemia surveillance

Prior to widespread UK deployment of PCV7 in the 2006/07 season, *S. pneumoniae* belonging to its target serotypes accounted for around half (44.4–53.6% in each of the years 2001 to 2006 inclusive) of all the *S. pneumoniae* collected in the BSAC bacteraemia surveillance but these declined to 4.7% of isolates by 2013 and 2.0% in 2014. Serotype 14 was the most common type in 6 of the 7 years from 2001 to 2007, comprising 13–20% of all isolates (Table 1) and accounting for 61% of all erythromycin-resistant isolates. By 2013, however, serotype 14 had only a single representative (0.4%), and none in 2014. Other serotypes became relatively more frequent as the PCV7 types declined, notably 7F and 19A, whereas serotype 1 had been expanding since 2001. These three types are within the spectrum of PCV13 and have declined, with variable rapidity, following its replacement of PCV7 in 2010. A further PCV13 type, serotypes 3, shows much less evidence of decline, as also noted elsewhere [13].

**Table 1:** Ten most-represented pneumococcal serotypes in the British Society for Antimicrobial Chemotherapy bacteraemia surveillance, United Kingdom and Republic of Ireland, 2001–2014 (n = 3,206 isolates)

	Serotype (bold); number and proportion of isolates																													% for top 10^a^
Rank	1		2	3			4		5			6			7				8		9					10				
2001 (n = 227)	**14**		**8**	**9V**			**23F**		**3**			**4**	**6B**	**12F**	NA				NA		**1**			9F		NA				71.4%
	n = 36 15.9%		n = 23 10.1%	n = 20 8.8%			n = 17 7.5%		n = 15 6.6%			n = 11 4.8%			NA				NA		n = 9 4.0%					NA				
2002 (n = 220)	**14**		**9V**	**6B**	**19F**	**23F**	**NA**		**NA**			**1**			**22F**				**8**		**3**					**4**	**7F**		**6A**	69.5%
																										**19A**	**20**			
	n = 42 19.1%		n = 24 10.9%	n = 14 6.4%			NA		NA			n = 11 5.0%			n = 10 4.5%				n = 9 4.1%		n = 8 3.6%					n = 7 3.2%				
2003 (n = 239)	**14**		**9V**	**1**			**4**		**8**			**23F**			**3**		**19F**		**NA**		**6B**					**18C**				72.0%
	n = 34 14.2%		n = 30 12.6%	n = 21 8.8%			n = 17 7.1%		n = 16 6.7%			n = 13 5.4%			n = 11 4.6%				NA		n = 10 4.2%					n = 6 3.8%				
2004 (n = 241)	**14**		**1**	**19F**			**4**	**23F**	**NA**			**9V**			**8**				**3**		**7F**	**19A**			**22F**	**NA**				67.2%
	n = 37 15.4%		n = 24 10.0%	n = 16 6.6%			n = 14 5.8%		NA			n = 13 5.4%			n = 13 5.4%				n = 11 4.6%		n = 10 4.1%					NA				
2005 (n = 230)	**14**		**1**	**9V**			**23F**		**3**	**4**	**8**	**NA**			**NA**				**19F**		**7F**					**6B**				75.2%
	n = 35 15.2%		n = 30 13.0%	n = 21 9.1%			n = 18 7.8%		n = 14 6.1%			NA			NA				n = 10 4.3%		n = 9 3.9%					n = 8 3.5%				
2006 (n = 231)	**1**		**14**	**9V**			**23F**		**6A**			**4**	**6B**	**7F**	**NA**				**NA**		**8**		**18C**			NA				75.8%
	n = 36 15.6%		n = 29 12.6%	n = 22 9.5%			n = 16 6.9%		n = 15 6.5%			n = 13 5.6%			NA				NA		n = 9 3.9%					NA				
2007 (n = 216)	**14**		**1**	**9V**			**8**		**7F**			**23F**			**3**	**4**		**6A**	**NA**		**NA**					**12F**				77.3%
	n = 30 13.9%		n = 26 12.0%	n = 20 9.3%			n = 19 8.8%		n = 14 6.5%			13 6.0%			n = 12 5.6%				NA		NA					n = 9 4.2%				
2008 (n = 201)	**1**		**14**	**8**			**7F**	**22F**	**NA**			**9V**			**19A**				**3**		**20**					**4**		**23F**		73.6%
	n = 32 15.9%		n = 20 10.0%	n = 17 8.5%			n = 15 7.5%		NA			n = 14 7.0%			n = 13 6.5%				n = 9 4.5%		n = 7 3.5%					n = 6 3.0% =				
2009 (n = 211)	**7F**		**3**	**19A**			**8**		**22F**			**1**			**6A**				**14**		**12F**					**4**				71.6%
	n = 26 12.3%		n = 23 10.9%	n = 18 8.5%			n = 17 8.1%		n = 14 6.6%			n = 13 6.2%			n = 12 5.7%				n = 11 5.2%		n = 9 4.3%					n = 8 3.8%				
2010 (n = 249)	**19A**		**7F**	**1**			**8**		**33F**			**22F**			**14**				**3**		**6A**		**11A**			NA				68.7%
	n = 38 15.3%		n = 33 13.3%	n = 21 8.4%			n = 19 7.6%		n = 14 5.6%			n = 11 4.4%			n = 10 4.0%				n = 9 3.6%		n = 8 3.2%					NA				
2011 (n = 230)	**7F**		**19A**	**8**			**3**		**22F**			**1**			**23B**				**9N**	**15A**	**NA**					**12F**		**19F**		70.4%
	n = 29 12.6%		n = 28 12.2%	n = 22 9.6%			n = 19 8.3%		n = 18 7.8%			n = 14 6.1%			n = 9 3.9%				n = 8 3.5%		NA					n = 7 3.0%				
2012 (n = 229)	**7F**		**8**	**22F**			**19A**		**33F**			**12F**			**1**		**3**		**NA**		**6C**					**15A**				69.4%
	n = 29 12.7%		n = 27 11.8%	n = 25 10.9%			n = 20 8.7%		n = 12 5.2%			n = 11 4.8%			n = 10 4.4%				NA		n = 8 3.5%					n = 7 3.1%				
2013 (n = 235)	**7F**	**8**	**NA**	**22F**			**3**	**19A**	**NA**			**23A**			**12F**				**15A**	**33F**	**NA**					**1**	**11A**		**24F**	66.8%
	n = 36 15.3%		NA	n = 15 6.4%			n = 14 6.0%		NA			n = 10 4.3%			n = 9 3.8%				n = 8 3.4%		NA					n = 7 3.0%				
2014 (n = 247)	**8**		**22F**	**12F**			**15A**		**19A**			**3**			**7F**				**9N**	**24F**	**NA**					**10A**				71.7%
	n = 43 17.4%		n = 22 8.9%	n = 20 8.1%			n = 19 7.7%		n = 16 6.5%			n = 14 5.7%			n = 13 5.3%				n = 11 4.5%		NA					n = 8 3.2%				

NA: not applicable.

Green: covered by PCV7; yellow: additional types covered by PCV13; pink: not covered by any conjugate vaccine.

^a^ When there is a tie for tenth rank, only one of the tied serotypes is counted into the percentage total for the top 10.

Serotype 15A isolates were encountered in each year from 2010 and the serotype was in the top 10 from 2011 onwards, whereas previously the type was sporadic. Other types that had long been encountered at moderate to low prevalence also became more prominent, including serotypes 8, and (albeit with considerable year-on-year variation) 22F.

Triple resistance was seen in just 60/3,206 isolates (1.97%) throughout the period reviewed and its prevalence exceeded 10% only among isolates of serotypes 37 (2/3 isolates), 6B (13/90 isolates, 14.4%) and, most strikingly, 15A (13/50, 26.0%). Triple-resistant serotype 15A *S. pneumoniae* were received in every year from 2011, although never previously. This observation, along with increasing numbers of 15A isolates among PHE reference submissions (below), prompted the present analysis.

### Serotypes among British Society for Antimicrobial Chemotherapy respiratory isolates

Unlike those collected in the BSAC Bacteraemia Surveillance, *S. pneumoniae* from the BSAC Respiratory Surveillance were not routinely serotyped until 2013/14, when 15A proved to be the most frequent serotype (Table 2), comprising 34 9.1% of all 375 isolates collected, with a similar pattern in 2014/15, when 15A comprised 46/430 (10.7%) of isolates. What is more, 15A was one of only four serotypes (the others being 6B, 19A and 19F) where triple resistance was seen in over 10% of representatives. Overall, triple resistance was seen in 13/34 (38.2%) serotype 15A isolates vs 14/341 (4.1%) of all other isolates in 2013/14 (p < 0.001, logistic regression adjusted for clustering by centre); there was an even sharper difference, 24/46 (52.2%) vs 25/384 (6.5%) (p <0.001), in 2014/15.

**Table 2:** Major serotypes and associations with resistance among *Streptococcus pneumoniae* from the British Society for Antimicrobial Chemotherapy Respiratory Surveillance, United Kingdom and Republic of Ireland, 2013/14 and 2014/15 (n=805)

Serotype	October 2013 to September 2014			October 2014 to September 2015		
	**Count**	**% of total isolates**	**No (%) with triple resistance**	**Count**	**% of total isolates**	**No (%) with triple resistance**
15A	34	9.1	13 (38.2%)	46	10.7	22 (47.8%)
23B	26	6.9	1 (3.8%)	21	4.9	0
3	22	5.9	0	26	6.0	0
11A	21	5.6	1 (4.8%)	34	7.9	1 (2.9%)
23A	21	5.6	0	30	7.0	4 (13.3%)
22F	19	5.1	0	17	4.0	0
6C	18	4.8	0	12	2.8	0
19A	17	4.5	5 (29.4%)	14	3.3	4 (28.6%)
24F	16	4.3	0	12	2.8	1 (8.3%)
35F	14	3.7	0	14	3.3	0
10A	14	3.7	0	12	2.8	0
31	14	3.7	0	16	3.7	0
16F	12	3.2	1 (8.3%)	19	4.4	0
15B	11	2.9	0	3	0.7	0
17F	11	2.9	0	16	3.7	0
19F	11	2.9	3 (27.3%)	14	3.3	5(35.7%)
35B	11	2.9	0	18	4.2	0
8	10	2.7	0	12	2.8	1 (8.3%)
Other serotypes, with < 10 isolates in one or both years	73	19.4	3 (4.9%)^a^	85	(21.7)	10 (2.3%)^a^
PCV7 serotypes	17	4.5	NA	20	4.6	NA
PCV13 serotypes	63	16.8	NA	67	15.6	NA
Total	375	100	27 (7.2%)	430	100	49 (11.4%)

NA: not applicable; PCV: pneumococcal conjugate vaccine.

^a ^In 2013/14, three 6B isolates had triple resistance; the 10 'Other serotype' isolates with triple resistance in 2014/15 comprised three non-typeable, two 12F and single representatives of 6B, 7F, 9N 9V and 23.

Also notable was the fact that PCV7 serotypes accounted for only 17/375 (4.5%) of all the respiratory *S. pneumoniae* in 2013/14 and PCV13 types for just 63/375 (16.8%); corresponding figures in 2014/15 were 18/430 (4.3%) for PCV7 types and 68/430 (15.8%) for PCV13 types. The sole previous season when *S. pneumoniae* from the Respiratory Programme were typed was 2005/06, immediately before UK introduction of PCV7 [24]. Then, among 749 isolates, 312 (41.7%) belonged to PCV7 types and 450 (60.1%) to PCV13 types (assuming all serogroup 7 isolates belonged to serotype 7F) whereas 36 (4.8%) belonged to serogroup 15, which was not split to its component (15A/B/C/F) serotypes. The declines in PCV7 types, PCV13 types, and the rise in serotype 15A (compared with all serotype 15 in 2005/06) were all highly significant (p < 0.001, logistic regression adjusted for clustering by centre).

### Serotype trends, Public Health England invasive isolate surveillance

PHE surveillance of invasive *S. pneumoniae* indicated dramatic changes in serotype prevalence over time, as reviewed previously [7,13], with these paralleling the shifts seen for BSAC bacteraemia isolates. Specifically, in each of the years up to and including 2007, five or six of the top 10 serotypes were PCV7 types, with serotype 14 the most abundant (Table 3), as in the BSAC series (Table 1). After 2007, PCV7 types declined, with none in the top 10 after 2009. Several of the additional types covered by PCV13, notably 3, 6A, 7F and 19A, became relatively more prominent from 2006 until 2011 while serotype 1, also a PCV13 additional type, was prominent even before 2011. Except for serotype 3, which showed little convincing trend, these additional PCV13 types declined in rank after 2010/11, with the peaking of serotypes 1 and 19A being especially marked.

**Table 3:** Predominant serotypes among *S. pneumoniae* serotyped by the Respiratory and Vaccine Preventable Bacteria Reference Unit, Public Health England from invasive infections, 2005–2014 (n = 45,645)

	Serotype (bold) number and proportion of isolates										Proportion for top 10
Rank	1	2	3	4	5	6	7	8	9	10	
2005 n = 4,662	**14**	**1**	**8**	**9V**	**4**	**23F**	**3**	**6B**	**7F**	**19F**	73.3%
	701	528	357	333	327	271	250	248	208	195	
	15.0%	11.3%	7.7%	7.1%	7.0%	5.8%	5.4%	5.3%	4.5%	4.2%	
2006 n = 4,857	**14**	**1**	**9V**	**8**	**23F**	**4**	**3**	**6B**	**7F**	**19F**	72.1%
	660	611	337	321	300	288	268	256	249	210	
	13.6%	12.6%	6.9%	6.6%	6.2%	5.9%	5.5%	5.3%	5.1%	4.3%	
2007 n = 4,673	**1**	**14**	**9V**	**8**	**7F**	**3**	**4**	**6A**	**23F**	**6B**	69.1%
	583	449	351	348	316	278	238	237	231	197	
	12.5%	9.6%	7.5%	7.4%	6.8%	5.9%	5.1%	5.1%	4.9%	4.2%	
2008 n = 4,978	**1**	**7F**	**8**	**3**	**22F**	**19A**	**6A**	**14**	**9V**	**23F**	66.4%
	592	474	372	359	328	307	239	238	206	189	
	11.9%	9.5%	7.5%	7.2%	6.6%	6.2%	4.8%	4.8%	4.1%	3.8%	
2009 n = 5,000	**7F**	**1**	**19A**	**3**	**22F**	**8**	**6A**	**12F**	**14**	**33F**	67.7%
	553	501	490	438	423	393	189	148	131	118	
	11.1%	10.0%	9.8%	8.8%	8.5%	7.9%	3.8%	3.0%	2.6%	2.4%	
2010 n = 4,881	**7F**	**19A**	**1**	**3**	**8**	**22F**	**33F**	**6C**	**12F**	**11A**	69.9%
	675	640	445	362	362	361	164	161	139	102	
	13.8%	13.1%	9.1%	7.4%	7.4%	7.4%	3.4%	3.3%	2.8%	2.1%	
2011 n = 4,549	**7F**	**19A**	**8**	**1**	**3**	**22F**	**12F**	**33F**	**6C**	**15A**	71.8%
	665	538	424	391	382	348	139	131	126	124	
	14.6%	11.8%	9.3%	8.6%	8.4%	7.7%	3.1%	2.9%	2.8%	2.7%	
2012 n = 4,092	**7F**	**8**	**19A**	**22F**	**3**	**1**	**15A**	**33F**	**6C**	**12F**	68.2%
	485	456	369	357	276	243	176	155	148	125	
	11.9%	11.1%	9.0%	8.7%	6.7%	5.9%	4.3%	3.8%	3.6%	3.1%	
2013 n = 3,995	**8**	**7F**	**22F**	**19A**	**3**	**15A**	**12F**	**1**	**24F**	**33F**	66.4%
	545	415	320	293	274	203	174	153	141	134	
	13.6%	10.4%	8.0%	7.3%	6.9%	5.1%	4.4%	3.8%	3.5%	3.4%	
2014 n = 3,959	**8**	**12F**	**22F**	**3**	**19A**	**15A**	**7F**	**9N**	**33F**	**24F**	67.9%
	599	336	334	243	229	224	219	170	168	167	
	15.1%	8.5%	8.4%	6.1%	5.8%	5.7%	5.5%	4.3%	4.2%	4.2%	

PCV: pneumococcal conjugate vaccine.

Green: covered by PCV7.

Yellow: additional types covered by PCV13.

Pink: not covered by any conjugate vaccine.

99% of isolates are from England, Wales and Northern Ireland, with the remaining few from Scotland, Crown Dependencies, Republic of Ireland and elsewhere.

As in the BSAC series, serotype 15A first appeared in the top 10 in 2011. It then advanced to seventh rank by 2012 and sixth rank in both 2013 and 2014, accounting for 5.7% of isolates (224/3,959) in the latter year. Again, the proportion of resistance was striking: among the 330 tested, fully 104 (31.5%) of bloodstream 15A *S. pneumoniae* for all years pooled had triple resistance, whereas triple resistance rates for all other isolates that ever featured in the top 10 were under 12.5% (Table 4). Proportions of serotype 15A isolates, taking 2005–2014 pooled, rose with the patient’s age, from 1.3% in the 0–5 year age group to 1.4% in the 6–35 year age group, 0.6% in the 36–45 year age group, 1.7% in the 46–55, 56–65 and 66–75 year age groups, reaching 2.4% in the 76–85 year age group and 3.1% among the over-85-year-olds (p < 0.001). Triple resistance was represented among serotype 15A *S. pneumoniae* throughout the surveillance period reviewed, with proportions as follows: 2005, 0/3 isolates with triple resistance; 2006, 1/4; 2007, 2/10; 2008, 4/13; 2009 7/13; 2010, 18/34; 2011, 10/33; 2012, 15/50; 2013, 19/63 and 2014, 33/114.

**Table 4:** Proportions of isolates with triple resistance to penicillin, erythromycin and tetracycline among frequent serotypes of *Streptococcus pneumoniae* from blood and cerebrospinal fluid infections, Public Health England surveillance, 2005–2014 (n = 13,551)

Serotype	Total	Triple resistance	% Triple resistance
15A	330	104	31.5
6B	420	51	12.1
19F	401	45	11.2
19A	987	83	8.4
23F	360	15	4.2
24F	124	5	4.0
9V	562	19	3.4
14	1,145	27	2.4
6A	366	3	0.8
8	1,197	3	0.3
6C	205	2	1.0
9N	261	1	0.4
3	777	2	0.3
33F	239	2	0.8
1	1,195	1	0.1
22F	761	1	0.1
12F	474	1	0.2
4	334	0	0
7F	1,155	0	0
All others	2,258	0	0
All isolates and serotypes	13,551	469	3.5

99% of isolates are from England, Wales and Northern Ireland, with the remaining few from Scotland, Crown Dependencies, Republic of Ireland and elsewhere.

Serotypes that reached a top-10 ranking in any surveillance year in Table 3 are line-listed.

The isolates tested for antibiotic susceptibility and resistance (n = 13,551, annual range 1,159–2,966 p.a.) are a subset of those in Table 3 and comprise all isolates from hospitals that participate in the EARS-net surveillance along with those bloodstream isolates where the referring laboratory specifically sought susceptibility testing. Inclusion of the latter group may over-represent resistant organisms, although there is no reason why it should do so disproportionately within particular serotypes.

### Serotype trends, isolates referred to Public Health England for investigation of resistance

Between 2005 and 2014, 1,536 *S. pneumoniae* from respiratory, ear and eye infections were referred to PHE (Table 5) for investigation of unusual resistance. These submissions constitute a heavily biased sample and lack a denominator, but do provide a rolling snapshot of *S. pneumoniae* isolates that sending laboratories (mostly in England and Wales) consider to be concerning. Overall, 896/1,536 (58.3%) had triple resistance. In the earlier years members of serotypes 19F, 9V, 6B and 14 dominated, collectively accounting for 82.8–92.4% of referrals from 2005 to 2007, before declining from the start of the ‘PCV7 era’. Serotype 19A accounted for a growing proportion of referrals from 2005, peaking at 23.3% in 2011, while serotype 15A represented just 0–4% of submissions throughout the period 2005 to 2008 but thereafter increased progressively, becoming the most commonly referred serotype in 2012. In 2013, it accounted for 31/92 of all submissions where typing was undertaken, and for 31/107 in 2014. These proportions were greater than ever previously achieved by any other serotype. Fully 83.0% of serotype 15A isolates (137/165) had the triple resistance vs 68.9–80.0% among serotype 9V, 19A and 19F referrals, with lower proportions for other serotypes (Table 4).

**Table 5:** Predominant serotypes among respiratory, ear and eye isolates of *Streptococcus pneumoniae* received by the Public Health England Colindale reference service, 2005–2014 (n=1,536)

	Number of isolates of indicated serotype in year:										Grand total	No with triple resistance	% with triple resistance
	2005	2006	2007	2008	2009	2010	2011	2012	2013	2014			
Serotype 19F	15	31	41	50	55	35	23	14	12	14	290	232	80.0
Serotype 19A	2	10	15	27	45	44	28	17	8	17	213	169	79.3
Serotype 15A	0	4	3	8	23	22	17	26	31	31	165	137	83.0
Serotype 6B	5	18	39	35	25	13	4	7	3	2	151	104	68.9
Non-typeable rough	3	8	16	23	33	18	10	15	5	2	133	81	60.9
Serotype 9V	7	36	25	15	18	10	0	0	1	2	114	16	14.0
Serotype 14	9	18	21	6	11	11	4	2	3	1	86	30	34.9
Serotype 23F	7	9	6	16	16	9	3	2	1	3	72	35	48.6
Serotype 35B	1	3	4	9	7	6	9	4	7	4	54	9	16.7
No serotype data	1	2	0	0	0	6	1	1	5	26	42	22	52.4
Serotype 6A	0	3	5	5	3	6	1	2	0	1	26	7	26.9
Serotype 11A	0	0	1	1	4	3	3	5	2	4	23	12	52.2
Serotype 3	0	1	3	0	3	4	1	2	4	3	21	3	14.3
Serotype 1	0	0	1	2	3	5	1	1	1	0	14	1	7.1
Serotype 13	0	2	0	1	6	1	0	0	1	0	11	0	0.0
All other types^a^	8	5	5	9	16	17	15	10	13	23	121	38	31.4
Total	58	150	185	207	268	210	120	108	97	133	1,536	896	58.3
15A as % typed reference submissions^b^	0.0	2.7	1.6	3.9	8.6	10.8	14.3	24.3	33.7	29.0	11.0		

95% of isolates were from England, Wales and Northern Ireland with the remainder from Scotland, Crown Dependencies, Republic of Ireland or elsewhere.

^a^ Not accounting for > 10 isolates in total over the surveillance period.

^b^ Excludes ‘no data row’ above from denominator.

### Genomic sequencing and phenotypes of serotype 15A isolates

Genomic sequencing was performed on 156 serotype 15A *S. pneumoniae*. These represented a diversity of resistance patterns, and including 50 with triple resistance; a limitation was that all 156 sequenced isolates dated from 2013 and 2014. MLST types were deduced from the sequence data, and 78 (50%) of the isolates were identified as belonging to ST63 (n = 61) or its single or double locus variants (n = 17). All of these 78 ST63-related isolates were resistant to erythromycin (also clindamycin, not shown) and 49 (62.8%) had the triple resistance profile (Table 6). The macrolide and clindamycin resistance correlated with the consistent presence of *erm(B)* genes, as detected by WGS. All 78 ST63-related isolates were found also to carry the tetracycline-resistance determinant, *tet(M)*; those (n = 65, 83.3%) that expressed tetracycline resistance had the intact gene, whereas those (n = 13, 16.7%) that were tetracycline-susceptible (all of them classical ST63 isolates) had a deletion of two nucleotides at codon 339, generating a premature stop codon and thereby inactivating the gene. Most of the 49 isolates with triple resistance were susceptible to alternative agents: 37 remained susceptible to ampicillin, 47 to moxifloxacin, 48 to cefotaxime and all 49 to vancomycin, all based on EUCAST breakpoints. Sequence types (STs) 3811 (n = 19), 58 and its single locus variants (SLVs) (n = 21), and 73 and its SLVs (n = 11) were all heavily represented among the 78 ST63-unrelated serotype 15A isolates and, among all these, just one isolate had triple resistance and three or fewer were non-susceptible to any one of erythromycin, tetracycline or penicillin.

**Table 6:** Sequence types in relation to resistance of serotype 15A *Streptococcus pneumoniae* subjected to genomic sequencing (n = 156)

		Number (%) non-susceptible (intermediate or resistant)			
	n	Erythromycin	Tetracycline	Penicillin	Triple resistance
ST63	61	61 (100%)	48 (78.7%)	46 (75.4%)	35 (57.4%)
ST63 SLV and DLV	17	17 (100%)	17 (100%)	14 (82.4%)	14 (82.4%)
Other 15A^a^	78	2 (2.6%)	3 (3.8%)	1 (1.3%)	1 (1.3%)

SLV: single locus variant; ST: sequence type.

^a^  Includes 21 ST58 and SLVs, 19 ST3811, 11 ST73 and SLVs and 27 isolates belonging to sequence types with four representatives or fewer.

WGS data were available for a further 141 non-15A *S. pneumoniae*, predominantly investigated owing to multidrug resistance. Six had ST63-related profiles and these all had triple resistance; three expressed serotype 19F, one serotype 21 and one 23F; the final isolate was typed using antisera as serotype 20 but was predicted to be serotype 11A based on WGS; review suggests that the original serotype determination was in error. The association with 19F (a PCV7 serotype) is notable (see Discussion), but members of this serotype were highly variable in terms of ST; among a total of 25 serotype 19F isolates sequenced, 22 with triple resistance, we recorded 12 different known STs, along with two new variants. No single ST had more than four representatives.

## Discussion

Deployment of PCVs has had clear public health benefits. The incidence of invasive pneumococcal disease has been reduced not only in vaccinated children, but also in elderly adults, who benefit from herd immunity [31]. There is also evidence of impact on non-invasive disease: thus, PCV7 deployment in the UK in 2006 also was followed by a 19% reduction in hospital admissions for community-acquired pneumonia (CAP) among children aged < 2 years, reversing a rising trend that had persisted during the preceding decade [32]. A similar reduction was reported in Italy [33]. Moreover, a Cochrane review concluded that PCV7 reduced the incidence of acute otitis media in healthy vaccinated children, although with less impact for those with a history of the illness or deemed to be ‘high risk’ [34]. Lastly, active PCV13 vaccination was recently shown to achieve a 50% reduction in the incidence of bacteraemia and non-invasive pneumonia in elderly adults, again reflecting displacement of vaccine serotypes [35].

A limitation to this pattern of successes is, however, that the PCV vaccines cover only the most prevalent pneumococcal serotypes, leaving scope for expansion of other types. Deployment of PCV7 was followed by increased prevalence of serotype 19A isolates, many of them multidrug-resistant, and, although serotype 19A is now covered by PCV13, a niche may be created for yet further types. Internationally, several groups have remarked on the increased prevalence of multidrug-resistant serotype 15A and 35B isolates [14-21] and a recent PHE analysis of invasive pneumococcal infections, using the data series of Table 3, noted 15A to be among several serotypes now increasing in numbers and proportion in the UK [13]. The present analysis extends these findings, confirming that serotype 15A *S. pneumoniae* are of growing importance, as also shown (i) in the BSAC bacteraemia series (Table 1), which overlaps the PHE series but also includes Scotland and Ireland, (ii) the BSAC series LRTI (Table 2), which is the sole UK surveillance to test *S. pneumoniae* from their predominant disease setting, and (iii) among PHE reference submissions, which provide a rolling snapshot of resistance phenotypes causing concern to microbiologists at sending laboratories, which are predominantly in England, Wales and Northern Ireland, although with a few isolates received from elsewhere (Table 5). By 2013 and 2014, serotype 15A was consistently (i) among the top 10 serotypes in both the PHE and BSAC surveillances of invasive *S. pneumoniae* (Tables 1 and 3), (ii) was the top serotype among respiratory isolates (Table 2) and (iii) accounted for almost one third of all the *S. pneumoniae* sent for reference investigation as ‘unusually’ resistant. Critically, and unlike other rising pneumococcal serotypes (8, 10A, 11A, 12F, and 24F –see Tables 1, 3 and ref [13]) serotype 15A isolates were commonly resistant or non-susceptible to multiple antibiotics, including macrolides, clindamycin, tetracycline and penicillin. While none of the surveillances captures clinical outcomes, the fact that serotype 15A is rising in invasive infections implies that these organisms are virulent.

Around one third of serotype 15A isolates had ‘triple resistance’ (i.e. to macrolides and tetracycline together with intermediate penicillin resistance), a higher proportion than for other serotypes (Table 4). This proportion did not change substantially over time (although assessment is complicated by small total numbers of isolates in the earlier years), indicating that the serotype was gaining prominence both generally and as a resistant type, again implying that the surface polysaccharides of serotype 15A support virulence.

Triple resistance among serotype 15A isolates was strongly associated (p < 0.0001, Fisher’s exact of chi-squared tests) with ST63 and its variants and extremely rare among serotype 15A isolates belonging to other STs. This association between serotype 15A, ST63 and multidrug-resistance has been made by others too [18,36,37] and it was suggested by Frazao et al. [38] that multidrug-resistant ST63–15A organisms arose by type transformation of ST63 strains previously expressing the 19F capsular serotype. The present results provide very little support for this hypothesis. Although 3/22 multidrug-resistant serotype 19F *S. pneumoniae* examined were ST63 single- or double-locus variants, the remaining 19/22 belonged to diverse sequence types; moreover, ST63 alleles have been reported to be associated with other serotypes besides 19F and 15A, including serotype 8 in Spain [39], where it is suggested that they may have arisen via serotype switching of earlier Sweden 15A lineages [40].

In summary, the present findings imply that conjugate vaccines will face an ongoing game of ‘catch-up’, as new serotypes rise to prominence, and that expansion beyond a 13-valent formulation will be needed. They are pertinent also to the debate as to whether PCV13 should be adopted for prophylactic vaccination against pneumonia in the elderly, as is advocated based on recent positive trial results in the Netherlands [35]. Such positive findings must be set against the fact that PCV13 strains now account for less than 20% of community-onset pneumococcal pneumonias in the UK (Table 2).

While the rise of any new multidrug-resistant type is of concern for patient management, the ST63–15A *S. pneumoniae* had high level resistances only to macrolides, clindamycin and tetracyclines; MICs of penicillin mostly remained in the range 0.12 to 0.5 mg/L, and this level of ‘non-susceptibility’ is unlikely to compromise outcomes, except in meningitis. Susceptibility to moxifloxacin and cefotaxime remained near-universal, and ampicillin MICs were twofold below those of penicillin, remaining in the susceptible range and reversing the usual pattern for penicillin-non-susceptible *S. pneumoniae*, where ampicillin MICs mostly exceed those of penicillin. Treatment of infections therefore is unlikely to present especial problems, unless macrolides or tetracyclines are used alone, for example in beta-lactam allergic patients.
